# SARS-CoV-2 Nsp2 recruits GIGYF2 near viral replication sites and supports viral protein production

**DOI:** 10.1093/nar/gkaf674

**Published:** 2025-07-24

**Authors:** Jimi Kim, Youngran Park, Doil Yoon, Sunghan Lee, Haedong Kim, Ka-Yun Ban, Jihye Yang, Da-Eun Choi, Jeesoo Kim, Jong-Seo Kim, V Narry Kim

**Affiliations:** Center for RNA Research, Institute for Basic Science, Seoul 08826, Republic of Korea; School of Biological Sciences, Seoul National University, Seoul 08826, Republic of Korea; Department of Life Sciences, Gachon University, Seongnam 13120, Republic of Korea; Department of Health Science and Technology, GAIHST, Lee Gil Ya Cancer and Diabetes Institute, Incheon 21999, Republic of Korea; Center for RNA Research, Institute for Basic Science, Seoul 08826, Republic of Korea; School of Biological Sciences, Seoul National University, Seoul 08826, Republic of Korea; Center for RNA Research, Institute for Basic Science, Seoul 08826, Republic of Korea; School of Biological Sciences, Seoul National University, Seoul 08826, Republic of Korea; Center for RNA Research, Institute for Basic Science, Seoul 08826, Republic of Korea; School of Biological Sciences, Seoul National University, Seoul 08826, Republic of Korea; Center for RNA Research, Institute for Basic Science, Seoul 08826, Republic of Korea; School of Biological Sciences, Seoul National University, Seoul 08826, Republic of Korea; Department of Health Science and Technology, GAIHST, Lee Gil Ya Cancer and Diabetes Institute, Incheon 21999, Republic of Korea; Center for RNA Research, Institute for Basic Science, Seoul 08826, Republic of Korea; School of Biological Sciences, Seoul National University, Seoul 08826, Republic of Korea; Center for RNA Research, Institute for Basic Science, Seoul 08826, Republic of Korea; School of Biological Sciences, Seoul National University, Seoul 08826, Republic of Korea; Center for RNA Research, Institute for Basic Science, Seoul 08826, Republic of Korea; School of Biological Sciences, Seoul National University, Seoul 08826, Republic of Korea; Center for RNA Research, Institute for Basic Science, Seoul 08826, Republic of Korea; School of Biological Sciences, Seoul National University, Seoul 08826, Republic of Korea; Center for RNA Research, Institute for Basic Science, Seoul 08826, Republic of Korea; School of Biological Sciences, Seoul National University, Seoul 08826, Republic of Korea

## Abstract

The SARS-CoV-2 genome encodes 16 nonstructural proteins (Nsps), with Nsp2 being the least conserved and understood. This study highlights a crucial role for Nsp2 in the early phase of the viral life cycle, particularly its interaction with GIGYF2, which relocates near double-membrane vesicles (DMVs) and enhances viral protein production. Deletion of the Nsp2-coding region from the viral genome led to a drastic reduction in viral RNA synthesis early in infection (3–4 h after infection). Interactome analysis in virus-infected cells identified GIGYF2, a host-encoded translational regulation protein, as a key Nsp2 partner. This interaction was confirmed for both SARS-CoV-1 and SARS-CoV-2. Depletion of GIGYF2 or its cofactor ZNF598 phenocopied the replication defects observed with Nsp2 deletion, suggesting their critical roles in viral reproduction. Upon infection, GIGYF2 and ZNF598 relocate to areas near DMVs, viral replication sites. This relocation does not occur with the Nsp2-deleted virus, indicating Nsp2’s role in directing GIGYF2 to DMVs. Formaldehyde crosslinking and immunoprecipitation sequencing (fCLIP-seq) identified regions within viral RNAs that potentially interact with GIGYF2, including those encoding M and Orf6. Depletion of GIGYF2 resulted in decreased protein expression of M and Orf6. Our findings reveal the function of Nsp2 in supporting viral protein production by exploiting GIGYF2 as a host factor.

## Graphical Abstract

**Graphical Abstract:**
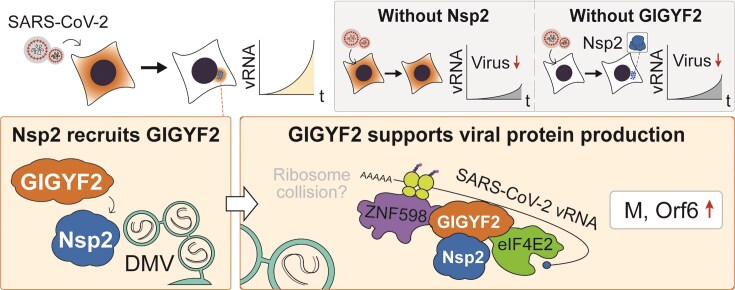

## Introduction

SARS-CoV-2 belongs to the genus *Betacoronavirus*, which consists of enveloped viruses with a long positive-sense single-stranded RNA genome (26 to 32 kb in length). Upon entering the host cell, the genomic RNA (gRNA) is translated into long polypeptides (Orf1a and Orf1ab) that are proteolytically processed into 16 fragments known as nonstructural proteins (Nsps) [1, 2]. Nsps support early stages of viral replication through both direct and indirect mechanisms. For instance, Nsp1 shuts down host translation and allows robust viral translation [3]. Nsp3, Nsp4, and Nsp6 collaborate to form specialized compartments called double-membrane vesicles (DMVs) by repurposing host endoplasmic reticulum (ER) membranes [4, 5]. Within DMVs, the viral RNA polymerase, Nsp12, synthesizes negative-sense viral RNAs, which in turn serve as templates for viral gRNAs and subgenomic RNAs (sgRNAs) [6, 7]. sgRNAs produce accessory proteins and structural proteins [1, 8, 9].

Nsp2 remains the least characterized of the 16 Nsp proteins. In SARS-CoV-1, deleting Nsp2 results in a 16-fold decrease in viral titer at 24 h post-infection (hpi), underscoring its importance in the viral life cycle, though its mechanism of action was enigmatic [10]. Studies involving ectopic expression and pull-down of Nsp2 protein from SARS-CoV-1 and SARS-CoV-2 have identified interacting partners, such as GIGYF2 and eIF4E2, known translational regulators [11–13]. Recent studies on SARS-CoV-2 Nsp2 have indicated its function in translation. Nsp2 overexpression resulted in an increase in cap-dependent translation as well as internal ribosome entry sequence-dependent translation [14]. Additionally, Nsp2 expression reduced the gene silencing activity of GIGYF2 and miRNA (microRNA)-induced silencing complex (miRISC) in reporter assays [15]. These results implicate Nsp2 in inhibiting translational repression induced by the GIGYF2–miRISC complex. However, this de-repression was observed in reporter assays without identifying the specific target gene(s) related to viral infection [15]. More recently, a seemingly contradictory finding has been reported, that is, Nsp2 strengthens the miRNA-mediated suppression in a manner dependent on GIGYF2 and eIF4E2 [16]. This Nsp2/GIGYF2/eIF4E2-mediated suppression is crucial for inhibiting type 1 interferon (IFN) translation during immune challenges like the poly(I:C) treatment or vesicular stomatitis virus infection. The repression was diminished in cells lacking GIGYF2 and eIF4E2 [17]. It is important to note that these findings are primarily from experiments with ectopic Nsp2 overexpression without actual virus infection, highlighting the need for comprehensive investigation into Nsp2’s role in the context of viral infection.

In this study, we investigated the molecular function of Nsp2 under the SARS-CoV-2 infection conditions. We discovered that Nsp2 relocates GIGYF2 in close proximity to the viral replication organelle and facilitates viral protein production.

## Materials and methods

### Cell culture

All cell lines used in this study were tested for mycoplasma contamination and confirmed to be negative. They were cultured in a humidified incubator at 37°C with 5% CO_2_. Vero (a gift from the International Vaccine Institute), Calu-3 (purchased from Korean Cell Line Bank), A549-ACE2 (a gift from B. tenOever at New York University), and HEK293T (a gift from S. Kim at Seoul National University) cell lines were grown in Dulbecco’s modified Eagle’s medium (DMEM) supplemented with 10% fetal bovine serum (FBS) (Welgene, LM 001-05). HCT-8 (purchased from the Korean Cell Line Bank) was cultured in RPMI with 10% FBS.

ACE2-overexpressing HEK293T cells were generated by transducing ACE2 constructs (Addgene, 161758) into HEK293T cells (Clontech, 632180). After transduction, the cells were subjected to antibiotic selection using hygromycin (Thermo Fisher Scientific, 10687010), followed by clonal selection. The overexpression of human ACE2 protein in HEK293T-ACE2 cells was confirmed by western blot analysis using an nti-ACE2 antibody (Thermo Fisher Scientific, MA5-32307).

### Plaque assay

For SARS-CoV-2 (a gift from the National Culture Collection for Pathogens, Korea National Institute of Health), Vero cells (2 × 10^5^) were seeded in 12-well plates. After 24 h, cells were washed with serum-free media and exposed to serially diluted virus in DMEM, gently rocking for 30 min. Following virus removal, the cells were covered with 1 ml of 0.8% low-melting agarose (Lonza, 50080) mixed with 1.6% serum-containing media and incubated at 37°C with 5% CO_2_ for 3 days to allow plaque formation. The cells were fixed with freshly prepared 4% formaldehyde in phosphate buffer edsaline (PBS) for 1 h at room temperature (RT). The agarose overlays were carefully removed using tap water. Plaques were stained with 1% crystal violet (Sigma–Aldrich, V5265) for 10 min and then rinsed with tap water multiple times. After drying the plates, plaques in wells with fewer than 50 plaques were counted to calculate the viral titer in plaque forming units (PFU)/ml. For OC43 virus (ATCC^®^ VR-1558D), HCT-8 cells were used following a similar protocol.

### siRNA transfection

Cells were seeded one day prior to transfection with a final concentration of 20 nM of short interfering RNA (siRNA) targeting the indicated genes. Transfection was performed using the Lipofectamine RNAiMAX (Thermo Fisher Scientific, 13778150) according to the manufacturer’s protocol. After a 24-h incubation, the cells were re-seeded into 12-well plates and subjected to a second round of siRNA transfection. Following another 24-h incubation, the cells were infected with SARS-CoV-2 virus at the indicated multiplicity of infection (MOI). Cells were harvested for RNA isolation using TRIzol reagent (Invitrogen, 15596-018) at designated time points. The sequences of the synthetic siRNAs used in this study are listed in Supplementary Table S1.

### Generation of knockout cell lines

To generate GIGYF2 knockout (KO) cell lines, Calu-3 and HEK293T-ACE2 cells were transfected with the pSpCas9 (BB)-2A-GFP-px458 plasmid (Addgene, 48138) and sgRNA (AAATTAGCAGATTATCGTTA, PAM sequence: CGG) targeting GIGYF2 using electroporation (Invitrogen Neon™ Transfection system) and Lipofectamine 3000 (Invitrogen, L3000001), respectively, following the manufacturer’s instructions. Following transfection, single-cell screening was performed, and KO was validated by Sanger sequencing and western blot analysis.

### Generation of cell lines stably expressing shRNAs

To generate cell lines stably expressing small hairpin RNAs (shRNAs) (shGIGYF2 and shNC), the lentiviral system was used as previously described [18]. The lentivirus was generated using the Lenti-X™ Packaging Single Shots kit (VSV-G) (Clontech, 631275). pLKO.1 lentiviral plasmid, carrying a puromycin resistance gene and an shRNA sequence, was transfected into Lenti-X 293T cells using Fugene^®^ HD (Promega, E2312) following the manufacturer’s instructions. Twenty-four hours after transfection, the supernatant was collected and filtered to remove cell debris. Calu-3 cells or A549-ACE2 cells were then infected with lentivirus-containing supernatant supplemented with 4 μg/ml of polybrene for 24 h. The supernatant was subsequently replaced with fresh media and incubated for another 24 h, followed by puromycin selection using media containing 2 μg/ml of puromycin.

### RT-qPCR and PCR

Virus-infected cells were treated with TRIzol reagent (Invitrogen, 15596-018). RNAs were isolated using the Direct-zol RNA miniprep kit (Zymo Research, R2052) and then reverse-transcribed with Primescript RTmix (Takara, R036A). The resulting complementary DNA (cDNA) was used as a template for qPCR and PCR. qPCR was performed using PowerSYBR Green (Applied Biosystems, 4367659) and analyzed with QuantStudio 5 (Thermo Fisher Scientific). PCR was conducted using Q5 High-Fidelity 2× Master Mix (NEB, M0492L) and analyzed with TapeStation (Agilent).

For messenger RNA (mRNA) expression analysis following reporter plasmid transfection, RNAs were isolated using the Direct-zol RNA miniprep kit with in-column DNase I digestion. Reverse transcription was performed using the PrimeScript RT reagent kit with gDNA Eraser (Takara, RR047A). qPCR was conducted using TB Green^®^ Premix Ex Taq™ II (Takara, RR82LR) and analyzed with the CFX384 Real-Time PCR Detection System (Bio-Rad). The list of primers is available in Supplementary Table S1.

### Generation of Nsp2-deleted and FLAG-Nsp2 SARS-CoV-2 cDNA

SARS-CoV-2 cDNA fragment clones, kindly provided by the Pei-Yong Shi lab (University of Texas Medical Branch, Galveston, TX, USA), were used to generate full-length cDNA as previously described [19]. Briefly, cDNA fragments were obtained from the plasmids by restriction enzyme digestion and gel extraction, and then assembled into a full-length cDNA genome using T4 DNA Ligase (NEB, M0202L). The ligated cDNA was purified using UltraPure Phenol:Chloroform:Isoamyl Alcohol (Thermo Fisher Scientific, 15593031) and verified on an agarose gel using ExcelBand XL 25 kb DNA Ladder, Broad Range (ExcelBand, ABIN5662613). To generate the Nsp2-deleted virus, the Nsp2 coding region was deleted from the F1 plasmid, positioning the Nsp1 and Nsp3 coding sequences adjacent to each other. For the FLAG-Nsp2 virus, the FLAG sequence with an additional 5′ sequence (GCATACACT) was inserted to induce PLpro-mediated cleavage between the C-terminus of Nsp1 and the N-terminus of FLAG (LNGG↓AYT). All plasmid sequences were validated by Sanger sequencing.

### RNA transcription, electroporation, virus production, and quantification

Virus RNA transcripts were synthesized *in vitro* using the mMESSAGE mMACHINE T7 Transcription Kit (Thermo Fisher Scientific, AM1344) in a 50 μl reaction containing 1 μg of DNA template at 37°C for 8 h. The transcribed RNA was purified using acid phenol:chloroform, followed by isopropanol precipitation at RT for 30 min, and then resuspended in nuclease-free water.

For SARS-CoV-2 production, the RNA transcripts were delivered to Vero cells stably expressing N, as previously described [19], with some modifications. Five micrograms of RNA transcripts were added to 2 × 10^6^ Vero-N cells suspended in 400 μl of Ingenio electroporation buffer (Mirus Bio, MIR 50114). The cells were electroporated using the Gene Pulser Xcell Eukaryotic System (Bio-Rad, 1652661) and then seeded in six-well plates with 2 ml of culture medium. The following day, the medium was replaced with fresh medium containing 2% FBS. After 3 days of incubation, the cells with medium were frozen at −80°C for a minimum of 3 h, thawed, and subjected to brief centrifugation for harvesting the virus-containing supernatant. The primary virus (P0) was further amplified in a 175 cm^2^ flask with 80% confluent Vero cells. The amount of virus was determined either by quantification of viral gene expression using reverse transcription quantitative polymerase chain reaction (RT-qPCR) or by a standard plaque assay. All virus work was performed in a Biosafety Level 3 (BSL-3) laboratory at the International Vaccine Institute.

### Immunoprecipitation–MS analysis

Virus-infected Calu-3 cells were crosslinked using 0.1% formaldehyde for 10 min and lysed with RIPA buffer (Thermo Fisher Scientific, 89901) containing 1× protease inhibitor (Calbiochem, 535140) and 1× phosphatase inhibitor cocktail (AG Scientific, P-1518). The lysate was incubated on ice for 20 min, treated with DNase I (Takara, 2270A) and RNase A (Thermo Fisher Scientific, EN0531) at 37°C for 30 min, and centrifuged at maximum speed for 20 min at 4°C. The supernatant was transferred to a new tube and incubated with antibody-conjugated agarose beads for 1 h at 4°C. Immunoprecipitates were washed stringently with RIPA buffer, incubated with 7 M urea (Sigma–Aldrich, U6504) for 30 min at 60°C, and subjected to FASP digestion using a 30 kDa MWCO Amicon filter (0.5 ml, Millipore, UFC503008) for mass spectrometry analysis [20]. To remove sodium dodecyl sulfate (SDS) from the filter unit, a series of washings with 200 μl of 50 mM ABC or 8 M urea and 15 min of 14 000 × *g* centrifugation were performed. Proteins were digested with 2% (w/w) trypsin at 37°C.

For peptide separation, a linear gradient with a flow rate of 300 nl/min was used on the nanoACQUITY UPLC (Waters) using an in-house packed trap column and a capillary analytical column with 3 μm Jupiter C18 particles. Then peptides were analyzed by the Orbitrap Eclipse spectrometer (Thermo Scientific) with the following parameters: m/z 350–1800 of precursor scan range, 1.4 Th of precursor isolation window, 30% of normalized collision energy (NCE) for higher-energy collisional dissociation (HCD), 30 s of dynamic exclusion duration, and 60k or 15k resolution at m/z 200 for full MS or MS/MS scan.

MS raw data files were searched by MaxQuant (version 1.6.2.3) with default settings (20 or 6 ppm of precursor ion mass tolerances for initial or main search) against the Uniprot proteome database at a protein false discovery rate (FDR) of < 1%, employing label-free quantification (LFQ) and match between runs. The Swiss-Prot human database (May 2019) and SARS-CoV-2 RefSeq open reading frame annotations (NC_045512.2, April 2020) supplemented with annotations for Orf3b/9b/9c were used. Nsps from Orf1a/1ab were annotated as separate entries.

SAINTexpress was used for IP-MS statistical enrichment analysis as outlined by Teo *et al.* [21]. For Nsp2 fCLIP–MS, proteins with average spectral counts ≥2, FDR < 0.01, and fold change ≥20 were defined as enriched over IgG control. Those with average spectral counts ≥2 and FDR < 0.05 were defined as enriched over the uninfected control. Proteins showing enrichment against both controls were identified as Nsp2 interactors. For GIGYF2 fCLIP–MS, proteins with average spectral counts ≥2, FDR < 0.01, and fold change ≥50 were defined as GIGYF2 interactors over IgG control. ER localization data were curated from Protein Atlas version 23.0 [22, 23].

### CRISPR screening data analysis

To construct a comprehensive dataset for comparative analysis with SARS-CoV-2 functional screen data, gene lists from 13 independent CRISPR screening studies were compiled [24–36]. The original publications’ criteria were primarily followed when identifying pro-viral factors from each study. If the FDR cutoff used in the previous studies was greater than 0.1, a more stringent cutoff (0.1) was applied to reduce the likelihood of false positives. When explicit cutoffs were not available or there were >200 proviral genes identified, additional selection criteria for MaGECK enrichment scores (<0.0001) or FDR-adjusted *P*-values (<.05) were adopted depending on the analytical methods used in the respective studies.

### Nsp2 sequence alignment

The amino acid sequences of Nsp2 from various human coronaviruses were aligned using ClustalW [37]. To visualize the alignment, a heatmap was generated, calculating similarity to the SARS-CoV-2 sequence based on the BLOSUM62 matrix [38]. For the heatmap, a score of −4 was assigned to sites of deletions or insertions.

### Western blotting

Cells were lysed using RIPA buffer supplemented with 1× protease inhibitor (Thermo Fisher Scientific, A32955) and 1× phosphatase inhibitor cocktail (AG Scientific, P-1518). The lysates were then centrifuged at maximum speed for 30 min at 4°C. The supernatants were transferred to new tubes and quantified by BCA protein assay kit (Thermo Fisher Scientific, 23227). An equal amount of protein from each sample was loaded on Novex™ Tris-Glycine protein gel (Invitrogen, XP08162BOX) with protein ladder, transferred to a PVDF membrane and incubated overnight with the following primary antibodies : anti-FLAG (Sigma–Aldrich, F7425), anti-GIGYF2 (Bethyl, A303-731A), anti-eIF4E2 (GeneTex, GTX103977), anti-GAPDH (Santa Cruz, sc-32233), anti-alpha-tubulin (Abcam, ab52866), anti-ZNF598 (Abcam, ab80458), anti-DDX6 (Bethyl, A300-461A), anti-CNOT9 (Proteintech, 22503–1-AP), anti-Membrane (Novus Biologicals, NBP3-05698), anti-Nsp1 (Genetex, GTX135612), anti-Nsp2 (Genetex, GTX135717), anti-Nsp3 (Abcam, ab283958), anti-GFP (Santa Cruz, sc-9996), and anti-β-actin (Santa Cruz, sc-47778). The membranes were washed with PBS-T (PBS containing 0.1% Tween 20) three times and subsequently incubated with anti-mouse or anti-rabbit HRP conjugated secondary antibodies (Jackson ImmunoResearch, 111-035-144). Signals were detected using the ChemiDoc XRS+ system (Bio-Rad) or Amersham ImageQuant 800 system (Cytiva Life Sciences).

### Co-Immunoprecipitation

HEK293T cells were transfected with specified plasmids using Lipofectamine 3000 (Thermo Fisher Scientific), following the manufacturer’s instructions. At 24 h post-transfection, cells were lysed in RIPA buffer (Thermo Fisher Scientific, 89901) supplemented with 0.2 μg/μl of RNase A (Thermo Fisher Scientific, EN0531), 1 mM DL-Dithiothreitol, 1× protease inhibitor, and 1× phosphatase inhibitor cocktail for 1 h on ice. The lysates were centrifuged at maximum speed for 30 min at 4°C, and supernatants were transferred to new tubes. For immunoprecipitation, 10 μl of anti-FLAG M2 affinity gels (Millipore, A2220) were added to 1.5 mg of the supernatant and rotated overnight at 4°C. The immunoprecipitates were washed three times with lysis buffer and subjected to western blotting analysis.

For immunoprecipitation with virus-infected HEK293T-ACE2 cells, the cells were treated with 0.1% formaldehyde for 10 min at RT, following the formaldehyde crosslinking and immunoprecipitation sequencing (fCLIP-seq) protocol. The cells were then lysed using the lysis buffer described in [39]. The lysates were incubated with anti-GIGYF2 antibody-conjugated agarose beads for 1 h at 4°C. After five stringent washes with wash buffer, the beads were treated with 7 M urea for 30 min at 60°C and subjected to western blot analysis.

### Immunofluorescence

Cells were seeded on sterile coverslips placed in the 12-well plates one day before either fixation (for uninfected cells) or virus infection (for infected cells). Both infected and uninfected cells were fixed using 4% paraformaldehyde for 30 min and then permeabilized with PBS containing 0.5% Triton X-100 for 7 min. After blocking with PBS-T containing 1% BSA for 1 h, the cells were incubated overnight at 4°C with primary antibodies: anti-dsRNA K1 (Jena Bioscience, RNT-SCI-10020200), anti-GIGYF2 (ProteinTech, 24790-1-AP), anti-FLAG [Sigma–Aldrich, F3165 (mouse) and F7425 (rabbit)], and anti-ZNF598 (Sigma–Aldrich, HPA041760). After stringent wash with PBS-T, cells were incubated for 1 h with ALEXA FLUOR™ conjugated secondary antibodies (Invitrogen, A21202, A11034, A11032, A21207). Coverslips were mounted on a slide glass with VECTASHIELD^®^ antifade mounting medium with DAPI (Vector Laboratories, H1200). Images were obtained using a Nikon ECLIPSE Ti2 confocal microscope and analyzed using FIJI [40].

### RNA-seq

RNAs were isolated from infected cells using the Direct-zol RNA miniprep kit (Zymo Research, R2052). Two hundred nanograms of RNAs were subjected to rRNA depletion using the Ribo-Zero kit (Illumina, TruSeq Stranded Total RNA Library Prep Human/Mouse/Rat, 20020596). An RNA-seq library was constructed following the manufacturer’s instructions using the MGIEasy RNA Directional Library Prep Set (MGI Tech). The library was sequenced on DNBSEQ-G50RS. Sequencing reads were mapped to the human-SARS-CoV-2 combined genome by STAR (v2.7.10b) with options considering non-canonical viral junctions as previously adopted [1, 41, 42]. Only uniquely mapped reads were subjected to gene-level quantification with featureCounts [43]. Read count normalization was conducted with the R/Bioconductor package DESeq2 (v1.38.0) [44]. IFN response genes were curated from the previous studies [45–47].

### fCLIP-seq

fCLIP-seq was conducted as previously described [39] with modifications to comply with BSL-3. In brief, Calu-3 cells were infected with SARS-CoV-2 at an MOI of 0.05 for 48 h. The infected cells were washed with PBS and treated with 0.1% formaldehyde (Pierce, 28906) for 10 min at RT. A 1.5 M glycine solution (Sigma–Aldrich, G7126) was used to quench the reaction. Following another wash with PBS, cells were lysed with a lysis buffer as previously described in [39]. Simply, the lysis buffer contains 1× D-PBS (Welgene, LB001-02), 0.1% SDS (Ambion, AM9823), 0.5% deoxycholate (Sigma–Aldrich, D6750), and 0.5% NP-40 (Sigma–Aldrich, 492016). The lysate was incubated with RNase A (Thermo Fisher Scientific, EN0531) for RNA fragmentation along with DNase I (Takara, 2270A) for 10 min at 37°C and then centrifuged at maximum speed for 10 min at 4°C. The supernatant was transferred to a new tube, precleared with sepharose beads, and then incubated with anti-GIGYF2 antibody-conjugated sepharose beads (A/G, GE Healthcare, 17-5138-01/17-0618-02) for immunoprecipitation. The immunoprecipitates were washed stringently, and RNAs were liberated with proteinase K (Roche, 3115879001) and 7 M urea at 60°C overnight, according to the guidelines approved by the International Vaccine Institute (IVI). RNAs were then purified, followed by DNase I (Takara, 2270A) treatment. The rRNAs were removed using the Ribo-Zero kit (Illumina, TruSeq Stranded Total RNA Library Prep Human/Mouse/Rat, 20020596). The purified RNAs were ligated to the 3′ adapter and subsequently resolved on a 6% denaturing urea-polyacrylamide gel electrophoresis (PAGE). RNA fragments within the 50–250 nt range were extracted. These 3′ adapter-ligated RNAs were then ligated to the 5′ adapter followed by reverse transcription and PCR amplification. The fCLIP-seq libraries were sequenced using the Illumina NovaSeq 6000 platform. The sequences of 3′ and 5′ adapters are available in Supplementary Table S1.

For fCLIP-seq analysis, the 5′ adapter and 3′ adapter sequences were trimmed from sequencing reads. Reads were aligned to the human-SARS-CoV-2 combined genome by STAR (v2.7.3a) with options considering non-canonical viral junctions as previously adopted [1, 41]. Gene annotations were retrieved based on the RefSeq database by the intersect tool in BEDTools (v2.25.0) and then rRNA and tRNA reads as well as multiply mapped reads were excluded for subsequent analyses [48]. Peak calling analysis was performed using Piranha (v1.2.1) with the -s -n -l -z 50 options [49]. Peaks were retained only if overlapping regions were detected with an adjusted *P*-value <.05 in both replicates. Among the overlapping peaks from the replicates, the one with the lowest adjusted *P*-value was selected as the representative. The enrichment for each peak was determined by comparing the relative number of reads passing through the peak region between the control and experimental samples.

### fCLIP RT-PCR

The initial experimental procedure is identical to fCLIP-seq preparation. Cells were crosslinked with 0.1% formaldehyde and lysed. Unlike fCLIP-seq, the lysates were treated with DNase I, but not RNase A. Subsequently, the lysates were incubated with anti-GIGYF2 antibody-conjugated magnetic beads (Invitrogen, 10002D/10004D), and RNAs were isolated. The isolated RNAs were reverse-transcribed using PrimeScript RT Master Mix (Takara, R036A). The resulting cDNA was then amplified with Q5 High-Fidelity 2× Master Mix (NEB, M0492L) and the results were analyzed using TapeStation (Agilent). The list of primers is available in Supplementary Table S1.

### IFN-β production assay

Calu-3 and HEK293T cells were seeded on a 12-well plate along with their GIGYF2 KO counterparts 1 day before IFN-β treatment. A concentration of 1 μg/ml poly(I:C) (Sigma–Aldrich, P9582) was treated to the cells for 6 h using Lipofectamine™ RNAiMAX Transfection Reagent (Invitrogen, 13778075) following the manufacturer’s instructions. The quantification of IFN-β in the media was conducted using the human IFN-β Quantikine ELISA kit (R&D Systems, DIFNB0) according to the manufacturer’s protocol.

### Tethering assay

One day before transfection, HEK293T cells were seeded in six-well culture plates and incubated at 37°C for 24 h. Following transfection with 1500 ng of pCK-SARS-CoV-2 Nsp2, 1500 ng of pCK-GIGYF2-TEV-HA-λN, and 250 ng of pmirGLO-9X boxB plasmids using Lipofectamine 3000, cells were incubated at 37°C for an additional 24 h. Luciferase assays were performed using the Dual-Luciferase Reporter Assay System (Promega, E1980) following the manufacturer’s protocol.

## Results

### Critical role of Nsp2 in the early phase of SARS-CoV-2 life cycle

**Figure 1.**
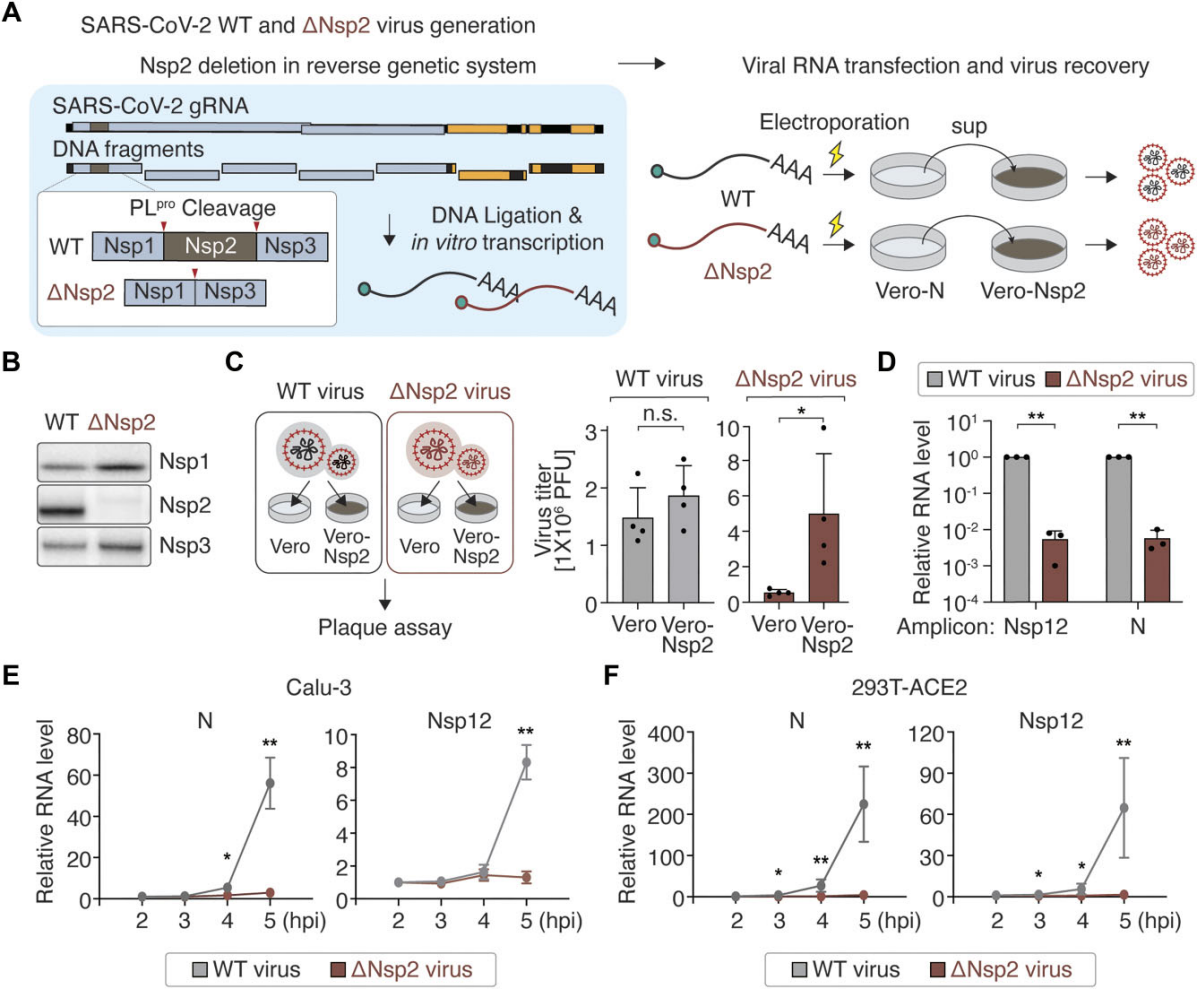
Critical role of Nsp2 in the early phase of SARS-CoV-2 life cycle. (**A**) Schematic of wild-type (WT) and Nsp2-deleted (ΔNsp2) SARS-CoV-2 generation. SARS-CoV-2 DNA fragments were used to generate the DNA encoding the full-length gRNA. For the ΔNsp2 virus generation, the Nsp2 sequence is removed from the first fragment. The proteolytic cleavage sites are indicated with arrowheads. The ligated DNAs were then transcribed *in vitro*. The full-length RNAs were subsequently electroporated into N-expressing Vero (Vero-N) cells to produce viruses. These viruses are amplified in Nsp2-expressing Vero (Vero-Nsp2) cells. (**B**) Expression of viral proteins, Nsp1, 2, and 3, in Vero cells infected with WT or ΔNsp2 SARS-CoV-2. (**C**) Schematic of viral MOI measurement using parental and Vero-Nsp2 cells. The WT and ΔNsp2 SARS-CoV-2 virus titers were measured by plaque assay using WT and Nsp2-expressing Vero cells. Data are shown as mean ± standard deviation (SD) (*n* = 4). One-sided Student’s *t*-test. (**D**) Relative RNA levels of Nsp12 and N following WT or ΔNsp2 SARS-CoV-2 infection. Calu-3 cells were infected with the indicated virus (MOI = 0.05). The RNAs were extracted at 24 hpi and subjected to RT-qPCR. Data are shown as mean ± SD (*n* = 3). One-sided paired *t*-test. (**E**, **F**) Time-course measurement of relative RNA levels of N and Nsp12 following WT or ΔNsp2 SARS-CoV-2 infection. Calu-3 cells (E) or 293T-ACE2 cells (F) were infected with the indicated virus (MOI = 1). The RNAs were extracted at the indicated time point post-infection and subjected to RT-qPCR. Data are shown as mean ± SD (*n* = 3). *P < .05, **P < .01, One-sided Student’s *t*-test.

To examine the role of Nsp2, we engineered the viral genome to generate an Nsp2-deleted virus (Fig. 1A). The DNA encoding the full-length viral genome was first constructed by ligation-based assembly using seven contiguous DNA fragments flanked by restriction sites (Fig. 1A and Supplementary Fig. S1A and B) [50]. The ligated DNA was then used for *in vitro* transcription to synthesize full-length viral gRNA, which was electroporated into cells to produce infectious viruses. As nucleocapsid (N) expression is known to enhance the infectivity of SARS-CoV-2 RNA transcripts [50], we utilized Vero cells stably expressing N protein (“Vero-N”) for gRNA transfection.

A mutation was introduced to the first DNA fragment (F1) such that the whole Nsp2-coding region was removed while maintaining the proteolytic cleavage site (Fig. 1A and Supplementary Fig. S1A). This way, we could successfully ablate Nsp2 without affecting Nsp1 and Nsp3, as confirmed by western blotting (Fig. 1B). To assess the impact of Nsp2 deletion on viral replication, we first amplified both WT and Nsp2-deleted (△Nsp2) viruses and measured their titers in Vero cells or in modified Vero cells stably expressing Nsp2 (“Vero-Nsp2”) (Fig. 1C and Supplementary Fig. S1C). The WT virus replicated comparably in both cell lines. In contrast, the Nsp2-deleted virus showed enhanced viral titer in Vero-Nsp2 cells compared with parental Vero cells, indicating that the ectopically expressed Nsp2 protein complemented the Nsp2 mutant virus. Next, these viral stocks were used to infect Calu-3 cells at an MOI of 0.05. Viral RNA levels were measured by RT-qPCR amplifying the Nsp12 and N regions, which served as indicators of gRNA and total viral RNAs (gRNA + sgRNAs), respectively. Viral RNA levels from the Nsp2-deleted virus were lower compared to those from WT virus at 24 hpi (Fig. 1D), indicating the importance of Nsp2 in the SARS-CoV-2 life cycle.

Notably, a time-course single-cycle experiment in Calu-3 cells showed that the Nsp2-deleted virus synthesizes viral RNAs at a substantially slower rate (Fig. 1E). The defect can be seen as early as 4 hpi. It takes ∼8 h to complete the SARS-CoV-2 life cycle, with structural and accessory genes starting to be detected at 4 hpi [51]. We also observed at least a 60-fold difference between WT and Nsp2-deleted virus using a HEK293T cell line stably expressing ACE2 (293T-ACE2) at 5 hpi (Fig. 1F). These findings highlight the critical role of Nsp2 in the early stages of SARS-CoV-2 replication.

### GIGYF2 is a functionally relevant partner of SARS-CoV-2 Nsp2

**Figure 2.**
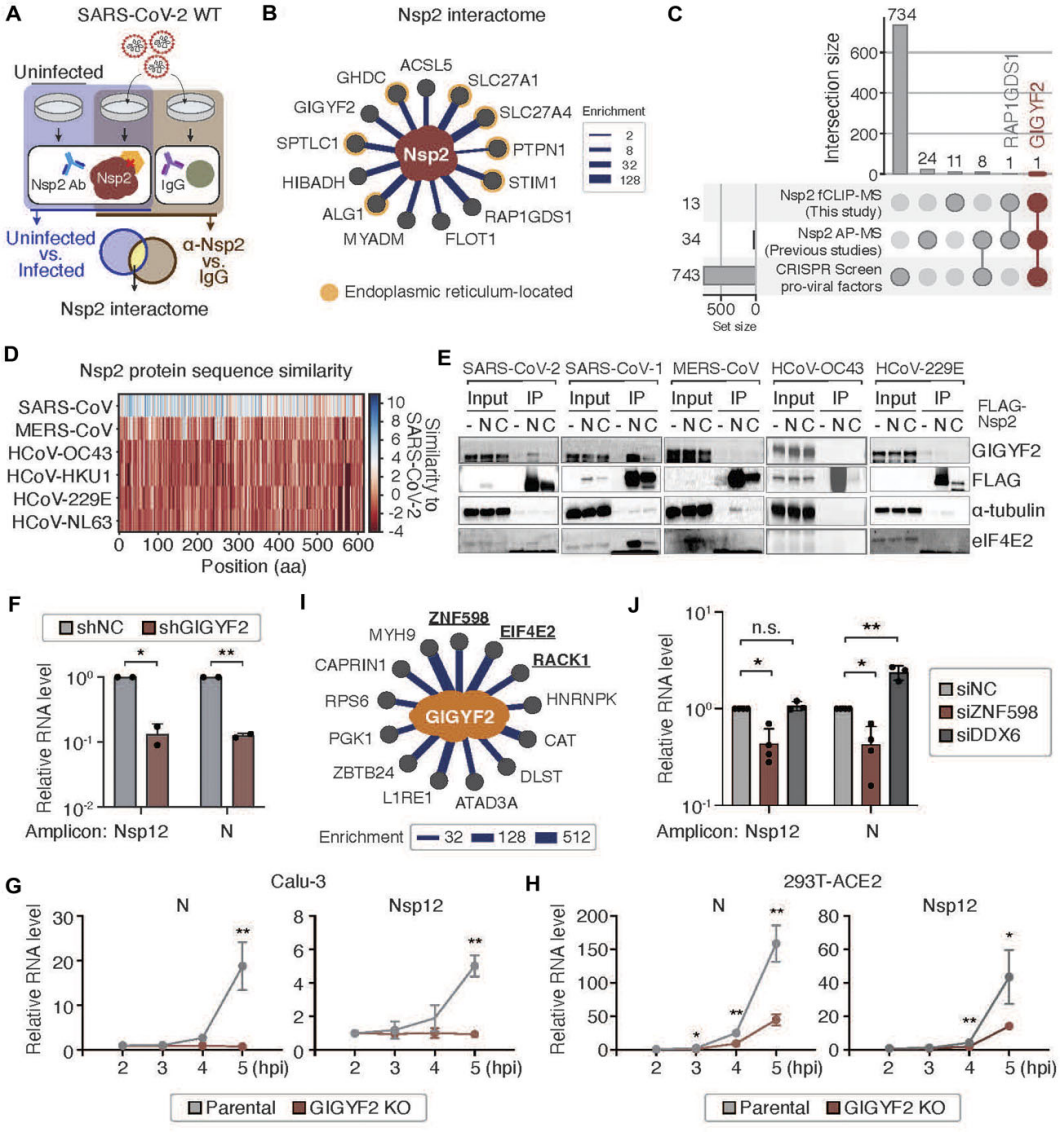
GIGYF2 is a functionally important binding partner of SARS-CoV-2 Nsp2. (**A**) Schematic of Nsp2 interactome study in Calu-3 cells infected with SARS-CoV-2 (MOI = 0.05). Cells were harvested at 48 hpi following formaldehyde crosslinking. Immunoprecipitation (IP) was carried out using (i) an anti-Nsp2 antibody in both uninfected and infected cells and (ii) anti-Nsp2 and IgG antibodies in infected cells. (**B**) Protein–protein interaction network of Nsp2 with host proteins. Proteins exhibiting enrichment under both conditions were classified as Nsp2 interactors. The edges width indicates the degree of enrichment in anti-Nsp2 immunoprecipitation between infected and uninfected cells. ER localization data were curated from Protein Atlas. (**C**) UpSet plot illustrating the overlap of SARS-CoV-2 Nsp2 host protein interactome from our fCLIP–MS with the Nsp2 AP-MS data and pro-viral candidates from CRISPR KO screens. Each bar represents the number of proteins overlapping between the indicated datasets. (**D**) The protein sequence similarity of Nsp2 in various human coronaviruses with SARS-CoV-2 Nsp2. (**E**) Physical interaction between GIGYF2 and Nsp2 proteins of various human coronaviruses. HEK293T cells were transfected with untagged (-), N-terminal tagged (N), or C-terminal tagged (C) Nsp2 proteins and harvested 48 h after transfection. IP was performed in the presence of RNase A overnight using the indicated antibodies. (**F**) Relative RNA levels of Nsp12 and N in GIGYF2 knockdown Calu-3 cells following SARS-CoV-2 infection (MOI = 0.05). RNAs were extracted at 24 hpi for RT-qPCR. Data are shown as mean ± SD (*n* = 3). One-sided paired *t*-test. (**G**, **H**) Time-course measurement of relative RNA levels of N and Nsp12 in parental and GIGYF2 KO cells following SARS-CoV-2 infection. Parental and GIGYF2 KO Calu-3 cells (G) or 293T-ACE2 cells (H) were infected with SARS-CoV-2 (MOI = 1). Data are shown as mean ± SD (*n* = 3). *P < .05, **P < .01, one-sided Student’s *t*-test. (**I**) Protein–protein interaction network of GIGYF2 with associated host proteins. IP was performed with anti-GIGYF2 antibodyor IgG, followed by LC-MS/MS (*n* = 2). Proteins with enrichment (anti-GIGYF2/IgG) equal to or greater than 50 were represented as core interactors. The width of the edges is proportional to enrichment. Host proteins associated with ribosome quality control (RQC) are highlighted. (**J**) Relative RNA levels of Nsp12 and N following depletion of GIGYF2 cofactors in SARS-CoV-2-infected cells. Calu-3 cells were transfected with siRNA targeting either ZNF598 or DDX6 for 48 h and then infected with SARS-CoV-2 (MOI = 0.05). RNAs were extracted at 24 hpi for RT-qPCR. Data are shown as mean ± SD (*n* = 3). One-sided paired *t*-test.

Recent interactome studies have identified interacting partners of SARS-CoV-2 Nsp2 [12, 13, 52]. However, because the prior studies relied only on ectopically overexpressed tagged Nsp2 in uninfected cells, their functional significance in the context of virus infection remained to be validated. To address the gap, we performed immunoprecipitation using an Nsp2-specific antibody and cell lysates from SARS-CoV-2-infected cells (Fig. 2A). Given that MHV Nsp2 is known to localize near DMVs, and DMV-associated proteins are insoluble in the lysates prepared with non-ionic detergents [53–56], we utilized a harsh lysis condition with ionic detergents in conjunction with formaldehyde crosslinking, followed by immunoprecipitation and mass spectrometry (fCLIP–MS). Two different controls were included to ensure specificity: (i) IgG and infected cell lysates and (ii) anti-Nsp2 antibody and uninfected cell lysates. The analysis identified 13 proteins significantly enriched over both controls (Fig. 2B, Supplementary Fig. S2A, and Supplementary Tables S2 and S3). Two proteins (GIGYF2 and RAP1GDS1) out of 13 candidates overlap with the previous datasets from affinity purification followed by mass spectrometry (AP–MS) with ectopically expressed SARS-CoV-2 Nsp2 [12, 13]. Thus, these two proteins are likely to interact robustly with Nsp2 independently of other viral components.

To pinpoint functionally relevant interactors, we compared our results with the data from previous CRISPR KO screens that aimed to identify host factors required for SARS-CoV-2 infection (Fig. 2C) [24–36]. Given the inherent uncertainty associated with antiviral hits in CRISPR screens using the cytopathic effect as a readout, we focused on the proviral hits. In this analysis, GIGYF2 emerged as the sole protein intersecting all the datasets. GIGYF2 is an RNA-binding protein that forms a complex with alternative cap-binding protein eIF4E2 (also known as 4EHP) and functions as a translation initiation repressor [57]. Notably, a recent comprehensive screen with Perturb-seq identified eIF4E2 as a critical host factor for SARS-CoV-2 [58]. Collectively, these data indicate that GIGYF2 is an Nsp2 interactor and may support SARS-CoV-2 viral replication.

Earlier interactome studies have detected GIGYF2 when using Nsp2 proteins from SARS-CoV-1 and MERS-CoV as baits [13]. Amino acid sequence comparison shows variability in Nsp2 across different human coronaviruses, with SARS-CoV-1 Nsp2 being the closest homolog to SARS-CoV-2 Nsp2 (Fig. 2D). To assess the conservation of the Nsp2-GIGYF2 interaction, we cloned Nsp2 proteins from five coronaviruses (SARS-CoV-2, SARS-CoV-1, HCoV-OC43, MERS-CoV, and HCoV-229E) and performed immunoprecipitation followed by western blot analysis (Fig. 2E). C-terminal FLAG tagging reduced protein expression relative to N-terminal tagging, likely due to decreased protein stability. We found that GIGYF2 and its cofactor eIF4E2 interact with Nsp2 from SARS-CoV-1 and SARS-CoV-2, confirming that the Nsp2-GIGYF2 interaction is conserved within SARS-CoVs. GIGYF2 was not co-precipitated with Nsp2 from the other tested coronaviruses under the conditions used, implying that their potential interaction might be weaker. These results suggest that GIGYF2 may serve as a conserved host factor at least for sarbecoviruses, potentially contributing to their replication through Nsp2-mediated mechanisms.

Next, we examined the impact of GIGYF2 on viral replication. To that end, we depleted GIGYF2 expression using shRNA in Calu-3 cells, and then infected them with SARS-CoV-2 (Fig. 2F and Supplementary Fig. S2B). GIGYF2 depletion resulted in more than a 70% reduction in viral RNA levels. Furthermore, we generated GIGYF2 KO cells by introducing CRISPR–Cas9 into Calu-3 and 293T-ACE2 cells and measured viral RNA levels in single-cycle infection conditions (Fig. 2G and H, and Supplementary Fig. S2B). In parental Calu-3 cells, viral RNA levels started to increase between 3 and 4 hpi, with N levels showing an ∼20-fold increase and Nsp12 levels increasing five-fold by 5 hpi (Fig. 2G). However, in GIGYF2 KO Calu-3 cells, we did not detect an increase even at 5 hpi. Likewise, SARS-CoV-2 replicated with a compromised rate in GIGYF2 KO 293T-ACE2 cells, compared to the parental 293T-ACE2 cells (Fig. 2H). The reduced replication kinetics in GIGYF2 KO cells resemble those of Nsp2-deleted SARS-CoV-2 (Fig. 1E and F). To compare SARS-CoV-2 with another human coronavirus, we depleted GIGYF2 with siRNAs in HCT-8 cells, which are susceptible to HCoV-OC43. In GIGYF2-depleted HCT-8 cells, HCoV-OC43 RNA levels were reduced by ∼25%, whereas in GIGYF2-depleted Calu-3 cells, SARS-CoV-2 RNA levels showed an ∼80% reduction (Supplementary Fig. S2C and D). This result suggests that SARS-CoVs may depend on GIGYF2 more strongly than the other coronaviruses. Collectively, our results demonstrate that GIGYF2, an Nsp2 interacting partner, is an important proviral host factor during the initial stages of SARS-CoV-2 replication.

When we performed fCLIP using anti-GIGYF2 antibody in the context of SARS-CoV-2 infection, Nsp2 was co-precipitated with GIGYF2 in 293T-ACE2 cells, validating the interaction between Nsp2 and GIGYF2 (Supplementary Fig. S2E). In Calu-3 cells, Nsp2 was not detected by fCLIP–MS, possibly because Calu-3 cells exhibit lower infectivity and produce lower levels of Nsp2. Thirteen host proteins were significantly enriched with anti-GIGYF2 antibody in Calu-3 cells, including eIF4E2, ZNF598, and RACK1 (Fig. 2I and Supplementary Table S4), consistent with earlier reports in uninfected cells [57, 59]. ZNF598 and RACK1 are known to be involved in the RQC pathway. Of particular interest is ZNF598, which plays a crucial role in recognizing ribosome collision and can engage the GIGYF2–eIF4E2 complex to tune translation initiation rate [60, 61]. To test its involvement in viral replication, we depleted eIF4E2 and ZNF598 in Calu-3 cells and infected the cells with SARS-CoV-2. We also examined the effect of depleting DDX6, another GIGYF2-interacting partner identified previously [61, 62]. Depletion of eIF4E2 and ZNF598 resulted in an ∼50% reduction of viral RNA levels, whereas depletion of DDX6 did not (Fig. 2J and Supplementary Fig. S2F). Taken together, these findings indicate that the GIGYF2–eIF4E2 complex plays a critical role, possibly in concert with ZNF598.

### SARS-CoV-2 Nsp2 recruits GIGYF2 to the vicinity of viral replication organelle

A previous study showed that Nsp2 of MHV is localized in the vicinity of DMVs—viral RNA synthesis sites [55, 56]. To examine whether SARS-CoV-2 Nsp2 is also localized near DMVs, we generated viruses that express the Nsp2 protein with a FLAG tag either at the N-terminus or the C-terminus (Supplementary Fig. S3A). These viruses enable biochemical assays and imaging in a biologically relevant context. Of note, the virus with the C-terminal FLAG tag showed replication defects (Supplementary Fig. S3B), which is in line with diminished Nsp2 expression observed in the presence of the C-terminal tag (Fig. 2E). Thus, we employed the virus with an N-terminal FLAG tag (“FLAG-Nsp2”) for subsequent assays. FLAG-Nsp2 protein was found to be enriched near double-stranded RNA (dsRNA), which was visualized by using K1 monoclonal antibody specifically recognizing dsRNA (Supplementary Fig. S3C). Since the major source of dsRNA in infected cells is viral replication intermediates, K1 staining represents active viral replication sites. Notably, when Nsp2 was expressed independently from a plasmid without viral infection, it diffused throughout the cytoplasm, suggesting that viral replication is required for Nsp2 enrichment near the nucleus (Supplementary Fig. S3D). We also observed that many ER-associated proteins are highly enriched in our Nsp2 interactome (Fig. 2B). As DMVs originate from ER membranes, these data collectively suggest that Nsp2 of SARS-CoV-2 is associated with DMVs.

**Figure 3.**
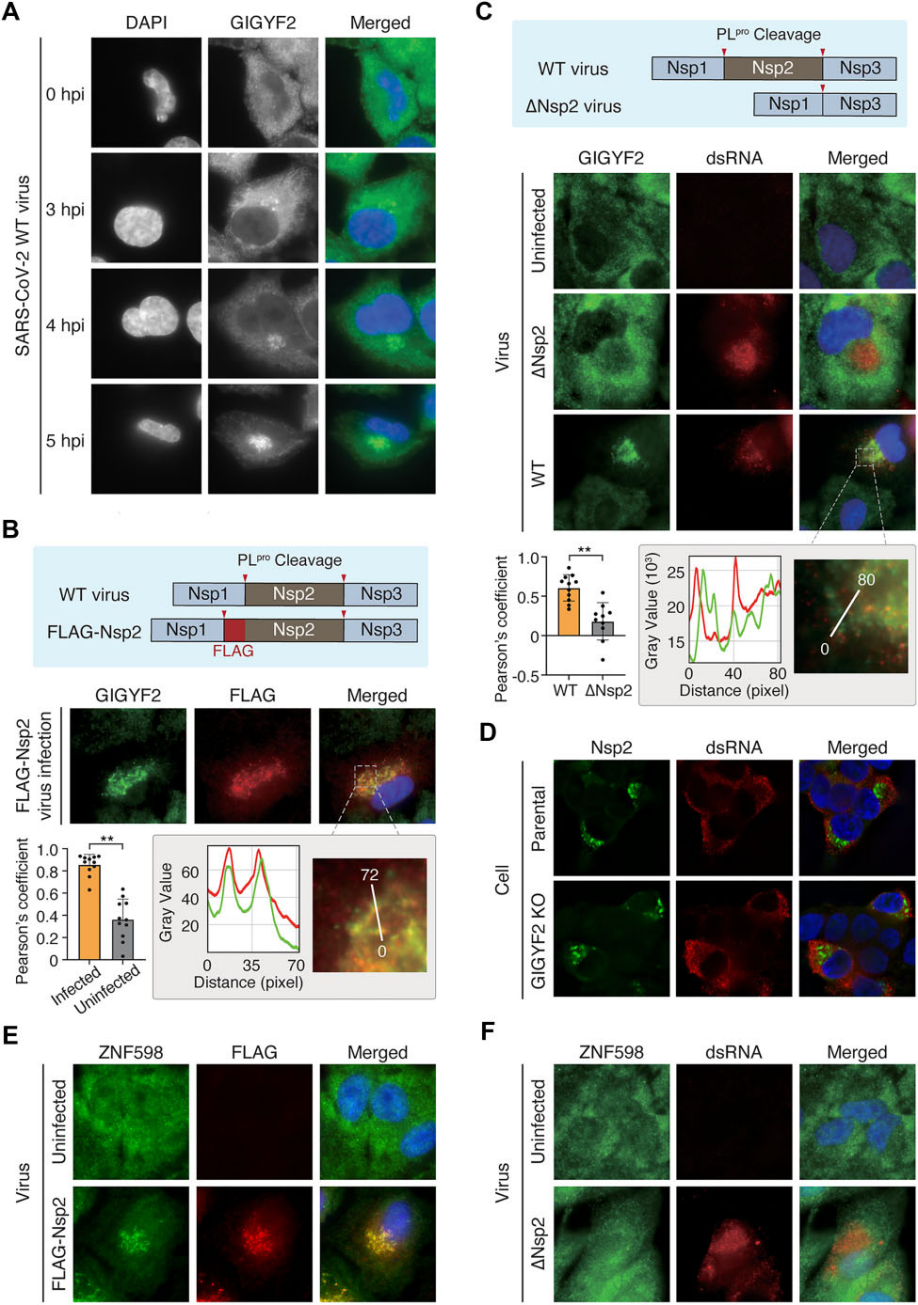
SARS-CoV-2 Nsp2 recruits GIGYF2 to the vicinity of viral replication organelle. (**A**) Time-dependent subcellular localization of GIGYF2 upon SARS-CoV-2 infection. A549-ACE2 cells were infected with SARS-CoV-2 (MOI = 10). Immunofluorescence staining of GIGYF2 (green) was performed at the indicated time points, along with DAPI staining (blue). (**B**) Subcellular localization of GIGYF2 and Nsp2 following infection of SARS-CoV-2 expressing FLAG-Nsp2. The upper panel depicts WT and FLAG-Nsp2 constructs, with proteolytic cleavage sites indicated by arrowheads. A549-ACE2 were infected with SARS-CoV-2 expressing FLAG-Nsp2 (MOI = 10). Immunofluorescence staining of GIGYF2 (green) and FLAG (red) was performed at 6 hpi, along with DAPI staining (blue). Overlap was quantified by Pearson’s colocalization coefficient (*n* = 11 images per sample). One-sided Student’s *t*-test. The bottom panel displays the fluorescence signal intensity profile across a marked line within the magnified regions, indicating the relative distribution of GIGYF2 and Nsp2. (**C**) Subcellular localization of GIGYF2 and dsRNA following WT or ΔNsp2 SARS-CoV-2 infection. The upper panel depicts WT and ΔNsp2 constructs, with proteolytic cleavage sites indicated by arrowheads. A549-ACE2 cells were infected with either WT or ΔNsp2 SARS-CoV-2 virus (MOI = 1 for WT and 10 for ΔNsp2). Immunofluorescence staining of GIGYF2 (green) and dsRNA (red) was performed at 24 hpi, along with DAPI staining (blue). Overlap was quantified by Pearson’s colocalization coefficient (*n* = 11 images for WT and *n* = 10 for ΔNsp2). One-sided Student’s *t*-test. The bottom panel displays the fluorescence signal intensity profile across a marked line within the magnified regions, indicating the relative distribution of GIGYF2 and dsRNA. (**D**) Subcellular localization of Nsp2 and dsRNA following SARS-CoV-2 infection in GIGYF2 KO cells. Parental and GIGYF2 KO 293T-ACE2 cells were infected with SARS-CoV-2 (MOI = 1). Immunofluorescence staining of GIGYF2 (green) and dsRNA (red) was performed at 24 hpi, along with DAPI staining (blue). (**E**) Subcellular localization of ZNF598 and Nsp2 following infection of SARS-CoV-2 expressing FLAG-Nsp2. A549-ACE2 cells were infected with SARS-CoV-2 expressing FLAG-Nsp2 (MOI = 0.22). Immunofluorescence staining of ZNF598 (green) and FLAG (red) was performed at 24 hpi, along with DAPI staining (blue). (**F**) Subcellular localization of ZNF598 and dsRNA following ΔNsp2 SARS-CoV-2 infection. A549-ACE2 cells were infected with ΔNsp2 SARS-CoV-2 (MOI = 5). Immunofluorescence staining of ZNF598 (green) and dsRNA (red) was performed at 24 hpi, along with DAPI staining (blue).

Next, we investigated the subcellular localization of GIGYF2 following virus infection. When we visualized endogenous GIGYF2 without infection, it was spread throughout the cytoplasm. However, upon SARS-CoV-2 infection, GIGYF2 formed a distinct cluster near the nucleus (Fig. 3A). Notably, GIGYF2 showed co-localization with Nsp2 (Fig. 3B, Supplementary Fig. S3F) and appeared to be in close proximity to dsRNA (Fig. 3C). The GIGYF2 cluster started to appear at 3 hpi and became more apparent at 4 hpi (Fig. 3A), which coincided with the viral replication defect observed with GIGYF2 KO cells (Fig. 2G and H). Importantly, when Nsp2-deleted SARS-CoV-2 was used instead of WT virus, the GIGYF2 localization did not change and remained diffuse throughout the cytosol (Fig. 3C). Therefore, the re-localization of GIGYF2 to DMVs is critically dependent on Nsp2. In contrast, Nsp2 is co-localized with dsRNA in both the parental and GIGYF2 KO Calu-3 cells, regardless of GIGYF2 ablation (Fig. 3D), suggesting that Nsp2 localizes to the vicinity of DMVs independently of GIGYF2. Of note, during HCoV-OC43 infection, the GIGYF2 cluster was not observed (Supplementary Fig. S3E), which is consistent with the notion that SARS-CoV-2 and SARS-CoV Nsp2 proteins may interact more strongly with GIGYF2 than the Nsp2 proteins of other coronaviruses.

Similar to the co-localization of GIGYF2 and Nsp2 near the DMVs, ZNF598 also co-localized with Nsp2 specifically upon viral infection (Fig. 3E). Notably, in the absence of Nsp2, ZNF598 was not found in proximity to dsRNA, suggesting that its relocalization to the DMVs is also Nsp2-dependent (Fig. 3F). Collectively, these data revealed that SARS-CoV-2 Nsp2 relocates GIGYF2 and ZNF598 near DMVs during viral replication.

### GIGYF2 interacts with viral RNA and ensures viral protein expression

**Figure 4.**
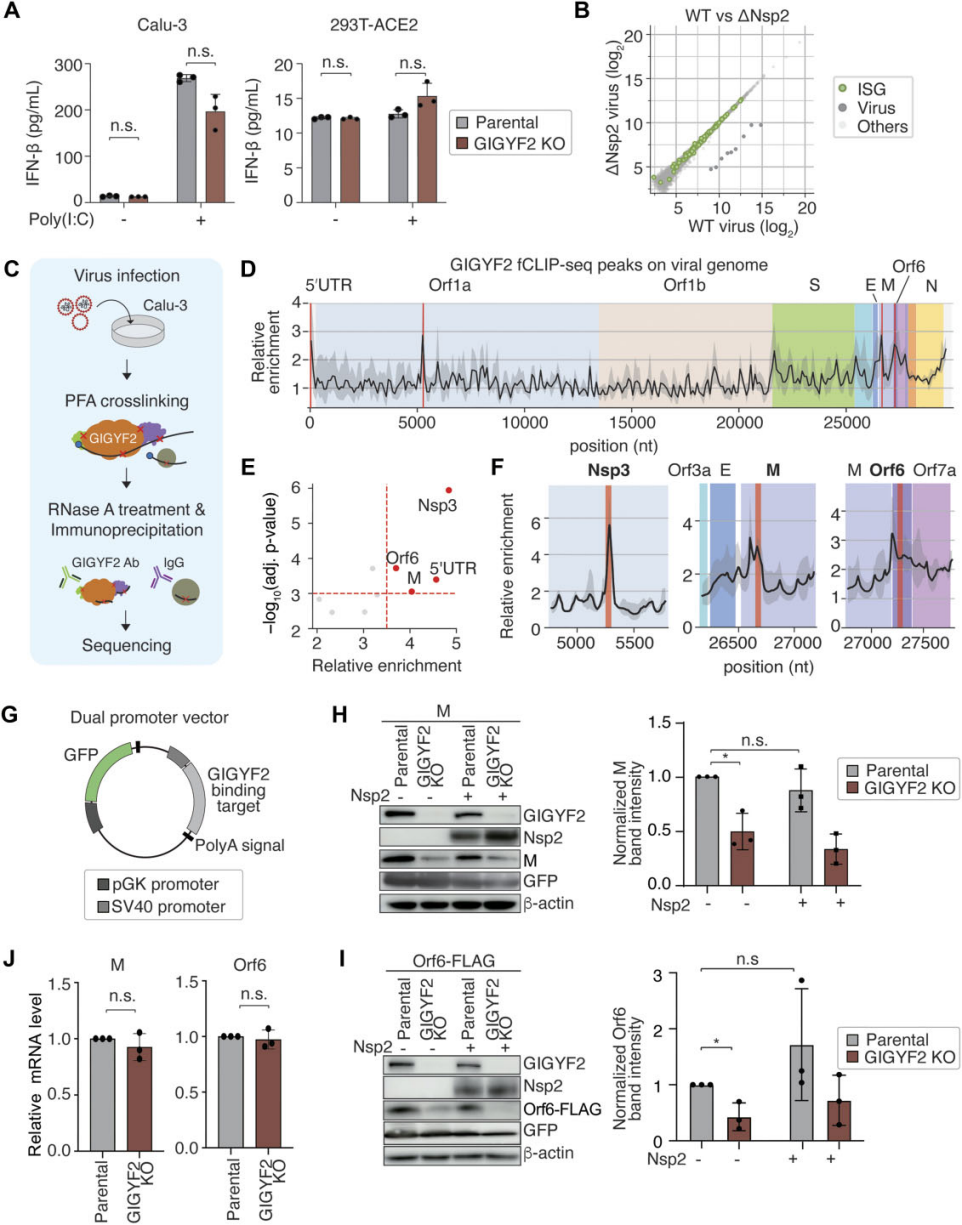
GIGYF2 interacts with viral RNA and ensures viral protein expression. (**A**) IFN-β measurement using ELISA. Parental and GIGYF2 KO variants of Calu-3 and 293T-ACE2 cells were treated with 1 μg/ml poly(I:C) for 6 h. ELISA was performed to quantify IFN-β levels. Data are shown as mean ± SD (*n* = 3). One-sided Student’s *t*-test. (**B**) A scatter plot representing the comparison of gene expression levels in Calu-3 cells at 5 hpi with ΔNsp2 (*y*-axis) and WT (*x*-axis) SARS-CoV-2 viruses (MOI = 0.05). The mean of normalized read counts in each condition is represented. Interferon-stimulated genes (ISGs) are marked in green. *n* = 2 for each condition. (**C**) Schematic of GIGYF2 fCLIP-seq after SARS-CoV-2 infection. Calu-3 cells were crosslinked with formaldehyde and subjected to RNase A treatment, followed by immunoprecipitation using IgG or anti-GIGYF2 antibody. (**D**) Enrichment of GIGYF2 fCLIP-seq reads across the SARS-CoV-2 genome (bin size = 100 nt, shift = 100 nt). Different genomic regions are color-coded, with regions showing significant peak enrichment highlighted in red. The line and shadow indicate the mean enrichment (anti-GIGYF2/IgG) and the standard deviation, respectively (*n* = 2). (**E**) Volcano plot showing the enrichment of GIGYF2 fCLIP-seq peaks in the viral genome. (**F**) Zoom-in view of selected GIGYF2 fCLIP-seq peaks in the viral genome (bin size = 50 nt, shift = 1 nt). The line and shadow indicate the mean enrichment (anti-GIGYF2/IgG) and the standard deviation, respectively (*n* = 2). (**G**) Schematic representation of a dual promoter vector designed for independent expression of GIGYF2 binding targets and green fluorescent protein (GFP). (H,I) Expression changes of GIGYF2 targets, M (**H**) and Orf6-FLAG (**I**), upon GIGYF2 depletion. 293T-ACE2 parental and GIGYF2 KO cells were transfected with the dual promoter vectors expressing the indicated GIGYF2 targets, with or without Nsp2. The original vector (pmiR-GLO dual luciferase) was used as negative control (NC). The band intensities of the GIGYF2 targets were measured using ImageJ software and normalized to GFP. Data are shown as mean ± SD (*n* = 3). *P < .05, one-sided paired *t*-test. (**J**) Relative mRNA expression of GIGYF2 targets (M, Orf6-FLAG) upon GIGYF2 depletion. 293T-ACE2 parental and GIGYF2 KO cells were transfected with the dual promoter reporter plasmids and subjected to RT-qPCR. Data are shown as mean ± SD (*n* = 3). One-sided paired *t*-test.

A recent study reported that Nsp2 reinforces GIGYF2-mediated translation repression of IFN-β. Specifically, Nsp2 expression augmented the silencing effect of GIGYF2 when tethered to a reporter mRNA and suppressed IFN-β production in a GIGYF2-dependent manner [17]. Moreover, depleting GIGYF2 increased IFN-β production following poly(I:C) treatment. However, in our experiments, GIGYF2 ablation did not significantly affect IFN-β production, with or without poly(I:C) treatment (Fig. 4A). Furthermore, no difference in ISG levels was observed between cells infected with the WT virus and those infected with the Nsp2-deleted virus (Fig. 4B and Supplementary Table S5). Thus, at least under the conditions used in our experiments, neither GIGYF2 nor Nsp2 influences ISG regulation. We also conducted tethering assays to evaluate the impact of Nsp2 on the expression of a luciferase reporter. While λN-tagged GIGYF2 reduced the expression of the reporter containing the boxB sequence, Nsp2 did not affect GIGYF2’s suppressive activity (Supplementary Fig. S4A). Taken together, our results suggest that GIGYF2 may promote SARS-CoV-2 replication via a mechanism distinct from the previously proposed immune regulatory function.

To understand the molecular mechanism by which GIGYF2 supports viral replication, we set out to identify GIGYF2 targets in the context of viral infection. GIGYF2 is known to interact with RNAs directly or indirectly via specific cofactors such as eIF4E2, ZNF598, and DDX6 [57, 63]. Thus, we employed fCLIP-seq, which was previously shown to capture both direct and indirect RNA targets (Fig. 4C) [64–66]. We confirmed that formaldehyde crosslinking effectively captures GIGYF2 cofactors (Supplementary Fig. S4B).

For fCLIP-seq analysis, we employed two types of controls: (i) immunoprecipitation with an IgG isotype control antibody and (ii) a size-matched input control, as each has inherent limitations. The IgG control suffers from low RNA yield, whereas the size-matched input control does not account for RNA abundance distortions introduced during immunoprecipitation. Our analysis revealed that GIGYF2 binds to both host and viral RNAs (Supplementary Tables S6–S8). Within the viral regions, the two controls identified distinct candidate targets: comparison with the IgG control showed enrichment of the 5′ UTR, Nsp3, M, and Orf6, whereas that with the size-matched input control highlighted N and the 3′ UTR (Fig. 4D–F and Supplementary Fig. S4C). To test the impact of GIGYF2 on these viral RNAs, we cloned the protein-coding regions into a dual promoter expression system, which co-expresses GFP from a separate promoter as a control (Fig. 4G). The plasmids were transfected into parental and GIGYF2 KO 293T-ACE2 cells. While GFP expression from the same plasmids remained unchanged upon GIGYF2 depletion, M and Orf6—targets identified using the IgG control—exhibited reduced expression, whereas N—a target identified using the input control—did not (Fig. 4H and I, and Supplementary Fig. S4G). Notably, the mRNA levels of M and Orf6 were unaffected in GIGYF2-depleted cells (Fig. 4J), suggesting that GIGYF2 enhances protein production at the translational level. Codon-optimized M and Orf6 also showed reduced protein levels in GIGYF2 KO cells (Supplementary Fig. S4D and E), implying that the nascent polypeptide chain may, at least partly, guide GIGYF2-mediated regulation for these proteins. Another target identified using the IgG control, Nsp3, also exhibited a reduction, albeit modestly, in the absence of GIGYF2 when expressed from the codon-optimized sequence (Supplementary Fig. S4F). Nsp3 expressed from its native sequence was below the detection limit, so we could not examine its dependence on GIGYF2. Codon-optimized Nsp9—a negative control not enriched in the fCLIP-seq data—was unaffected by GIGYF2 (Supplementary Fig. S4H).

GIGYF2 has previously been implicated in the regulation of human mRNAs encoding transmembrane proteins [60]. Notably, Nsp3, M, and Orf6—but not N—encode membrane-associated proteins, aligning with this function. Additionally, we observed a higher-than-expected representation of transmembrane protein-coding mRNAs among the top host transcripts identified by the IgG control (Supplementary Fig. S4I). Interactions between GIGYF2 and target RNAs were validated by fCLIP followed by RT-PCR (Supplementary Fig. S4J and K).

Lastly, to assess the role of Nsp2, we overexpressed it and found that Nsp2 did not influence M and Orf6 expression under the condition where DMVs were absent (Fig. 4H and I). Combined with our immunocytochemistry results (Fig. 3) and tethering experiments (Supplementary Fig. S4A), this suggests that while Nsp2 recruits GIGYF2 near DMVs to increase GIGYF2’s local concentration during infection, Nsp2 is not required for GIGYF2 activity *per se* under uninfected conditions.

## Discussion

**Figure 5.**
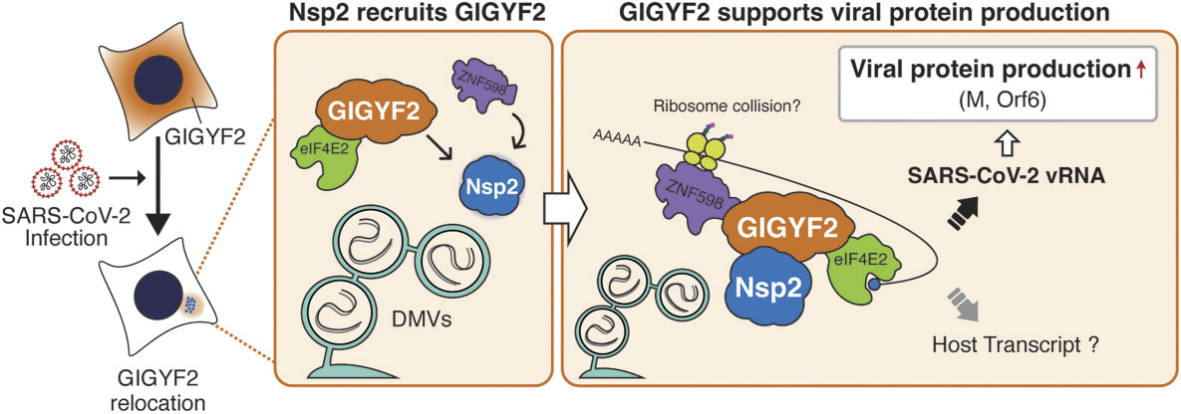
A proposed mechanism by which SARS-CoV-2 exploits host translational regulators. The model suggests that SARS-CoV-2 Nsp2 recruits GIGYF2-eIF4E2 and ZNF598 in proximity to DMVs, ensuring viral protein expressions, including M and Orf6.

In this study, we found a previously underappreciated role of Nsp2 in the SARS-CoV-2 life cycle. Deletion of Nsp2 from the genome results in a marked reduction of viral reproduction. Nsp2 physically interacts with GIGYF2 and induces its relocation close to viral RNA synthesis sites—DMVs. GIGYF2 then potentially associates with viral transcripts together with its cofactors, eIF4E2 and ZNF598, supporting viral protein production (see Fig. 5 for a model).

GIGYF2 has been reported to interact with RNAs from various viruses, including Ebola, Dengue, and Zika viruses, suggesting its broad role in viral regulation [67, 68]. Notably, our data demonstrate that SARS-CoV-2 Nsp2 relocates GIGYF2 from a diffuse cytosolic distribution to the vicinity of DMVs during infection. We speculate that this relocation allows GIGYF2 to swiftly bind newly synthesized viral mRNAs as they exit DMVs. GIGYF2 functions as a translation initiation repressor in a complex with eIF4E2, an alternative cap-binding protein competing with translation factor eIF4E for the 5′ m7G cap. eIF4E2 binds to a cap analog with 30 times lower affinity than eIF4E and can outcompete eIF4E only when highly concentrated [69–71]. Thus, the local enrichment of the GIGYF2/eIF4E2 complex near viral RNAs may help selectively tune the translation initiation of viral mRNAs.

Our fCLIP-seq data suggests that GIGYF2 binds to many transcripts, including SARS-CoV-2 RNAs, with particular enrichment in regions encoding transmembrane proteins, Nsp3, M, and Orf6. GIGYF2 facilitates viral protein production without altering RNA abundance, suggesting a regulatory mechanism at the translational step. This result was unexpected given the established role of GIGYF2 as a translation initiation suppressor. Recent studies have suggested that GIGYF2/eIF4E2 regulate transcripts experiencing ribosome collisions during translation [61, 72]. Intriguingly, it was also proposed that while prolonged ribosome collisions lead to degradation of mRNAs and nascent peptides, transient ribosome stalling may provide time for co-translational protein folding and membrane targeting [71, 73–76]. Notably, we observed that ZNF598, a ribosome collision detector, also localizes near DMVs via Nsp2 upon SARS-CoV-2 infection. Therefore, one can envision that the GIGYF2–eIF4E2–ZNF598 complex prevents excessive ribosome loading, ensuring proper folding and expression of viral membrane proteins. We observed the positive effect of GIGYF2 on the target viral protein production regardless of codon optimization. This implies that if ribosome pausing occurs on the viral transcript, it may be driven at least in part by amino acid sequences, consistent with previous studies [77–79]. Further investigation will be necessary to elucidate the detailed molecular mechanism by which GIGYF2/eIF4E2 regulates viral mRNA translation.

To investigate the role of GIGYF2 in viral replication, our study focused on the Nsp2-GIGYF2-viral transcript axis; however, we do not exclude the possibility that alternative pathways involving Nsp2 and GIGYF2 may also contribute. We observed that GIGYF2 also interacts with some host transcripts, suggesting that it may influence viral replication, in part, through the regulation of host genes. Additionally, we and others have identified multiple Nsp2-associated host proteins, including RAP1GDS1 [12, 13], which may further modulate the viral life cycle.

Furthermore, while our functional assays using a dual-promoter expression system demonstrated that depletion of GIGYF2 significantly affects the expression of viral M and Orf6 proteins, it remains possible that GIGYF2 may interact with and regulate additional viral targets as well. Given that formaldehyde crosslinking captures both direct and indirect interactions and that GIGYF2 forms a complex with multiple cofactors, our fCLIP-seq data likely reflect a complex network of GIGYF2-associated RNAs. Notably, the fCLIP-seq analysis using a size-matched input control revealed a distinct set of candidate targets compared to those enriched relative to the IgG control, highlighting the need to validate bona fide interactions. Further mechanistic and technical investigations will be required to fully understand how Nsp2 and GIGYF2 cooperate to promote efficient viral replication.

Our work underscores the importance of Nsp2 and GIGYF2 in viral infection. Nsp2 is a rapidly evolving protein with low sequence conservation across different human coronaviruses. Nevertheless, Nsp2’s interaction with GIGYF2 is conserved at least in SARS-CoV-1 and SARS-CoV-2, suggesting an evolutionary adaptation that imparts a novel function within the subgenus *Sarbecovirus*. Our study demonstrates that SARS-CoV-2 effectively exploits GIGYF2 and its cofactors through Nsp2 to ensure optimal viral protein expression, possibly by tuning translation.

## Supplementary Material

gkaf674_Supplemental_Files

## Data Availability

The mass spectrometry proteomics data underlying this article are available via ProteomeXchange with identifier PXD052138. The sequencing data underlying this article are available at the NCBI Gene Expression Omnibus (accession numbers: GSE269563 and GSE269564). Custom analysis codes are available at https://doi.org/10.5281/zenodo.15000087.
