# Supplementary material for: SARS-CoV-2 Nsp2 recruits GIGYF2 near viral replication sites and supports viral protein production
Source: Nucleic Acids Res. 2025 Jul 24;53(14):gkaf674. doi: 10.1093/nar/gkaf674 (PMC12288870; doi:10.1093/nar/gkaf674)
Supplement: gkaf674_Supplemental_Files [file gkaf674_supplemental_files.zip › NAR_Kim J. et al. Supplementary Figures with legends_2nd_final.pdf]

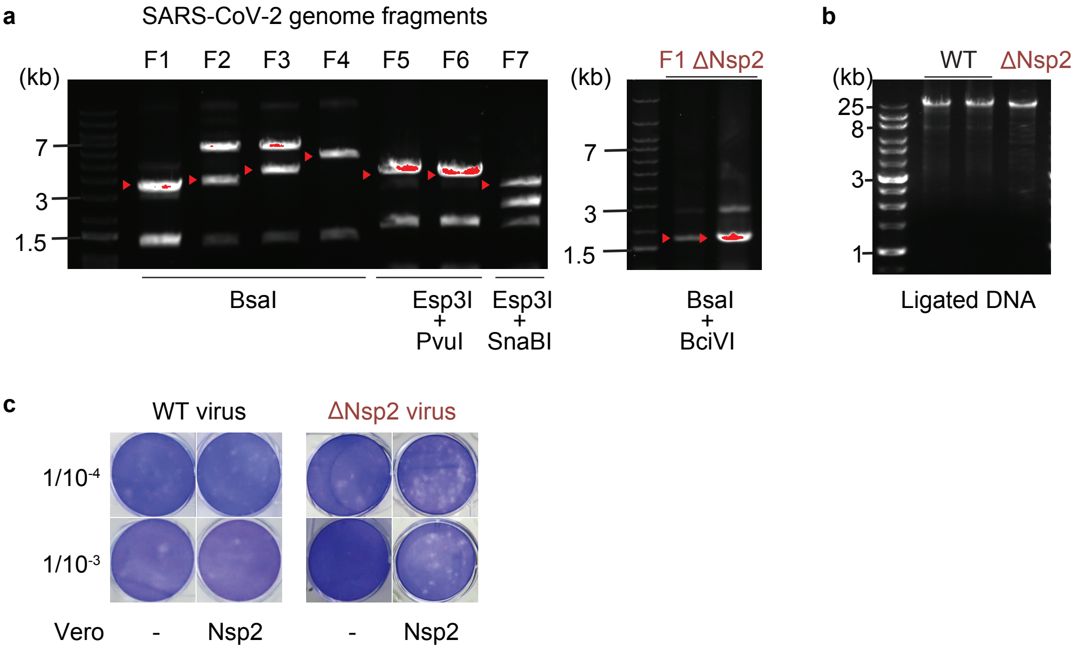

Supplementary Figure 1. Construction and functional assays of ΔNsp2 SARS-CoV-2

(a) Gel electrophoresis of DNA fragments encoding the SARS-CoV-2 genome. The plasmids containing each fragment were digested by indicated restriction enzymes and loaded onto 0.6 % agarose gels. Nsp2 is located within the first fragment (F1).

(b) Gel electrophoresis of the ligated DNA encoding the full-length SARS-CoV-2 genome (WT or ΔNsp2). The ligated products were loaded onto 0.8 % agarose gels.

(c) Plaque assay with WT or ΔNsp2 SARS-CoV-2 using parental or Vero-Nsp2 cells. The dilution factors used for the assay are indicated on the left.

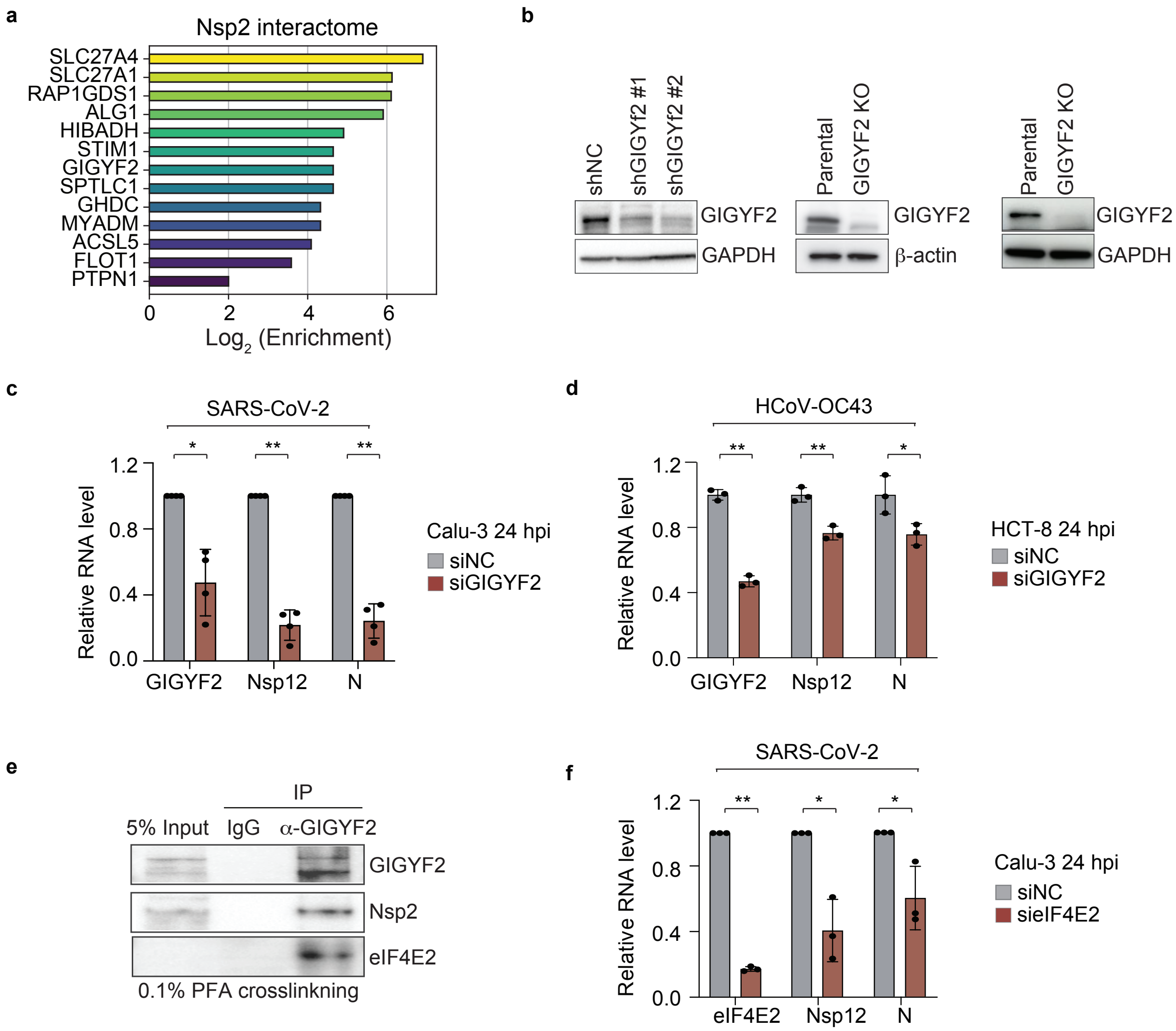

Supplementary Figure 2. GIGYF2 depletion and its impact on viral gene expression

(a) Horizontal bar plot showing the enrichment of Nsp2-interacting proteins.

(b) GIGYF2 expression level. GIGYF2 levels were measured in Calu-3 cells stably expressing either shNC or shGIGYF2 (left), Calu-3 parental and GIGYF2 KO cells (middle), and 293T-ACE2 parental and GIGYF2 KO cells (right). GAPDH and β-actin are shown as loading controls.

(c) Relative RNA levels of GIGYF2, Nsp12, and N in siRNA treated cells following SARS-CoV-2 infection. Calu-3 cells were transfected with siRNA targeting GIGYF2 or NC for 48 hr and then infected with SARS-CoV-2 (MOI = 0.05). RNAs were extracted at 24 hpi and subjected to RT-qPCR. Data are shown as mean ± s.d. (n = 3). Paired t-test.

(d) Relative RNA levels of GIGYF2, Nsp12, and N in siRNA treated cells following HCoV-OC43 infection. HCT-8 cells were transfected with siRNA targeting GIGYF2 or NC for 24 hr and then infected with HCoV-OC43 (MOI = 5). RNAs were extracted at 24 hpi and subjected to RT-qPCR. Data are shown as mean ± s.d. (n = 3). One-sided Student's t-test. NC, negative control.

(e) Interaction between GIGYF2 and SARS-CoV-2 Nsp2. 293T-ACE2 cells were infected with SARS-CoV-2 (MOI = 0.05) and harvested at 48 hpi following formaldehyde crosslinking. IP was performed in the presence of RNase A overnight using the indicated antibodies.

(f) Relative RNA levels of eIF4E2, Nsp12, and N in siRNA treated Calu-3 cells following SARS-CoV-2 infection.Calu-3 cells were transfected with siRNA targeting eIF4E2 or NC for 48 hr and then infected with SARS-CoV-2 (MOI = 0.05). RNAs were extracted at 24 hpi and subjected to RT-qPCR. Data are shown as mean ± s.d. (n = 3). *P < 0.05, **P < 0.01, One-sided paired t-test. NC, negative control.

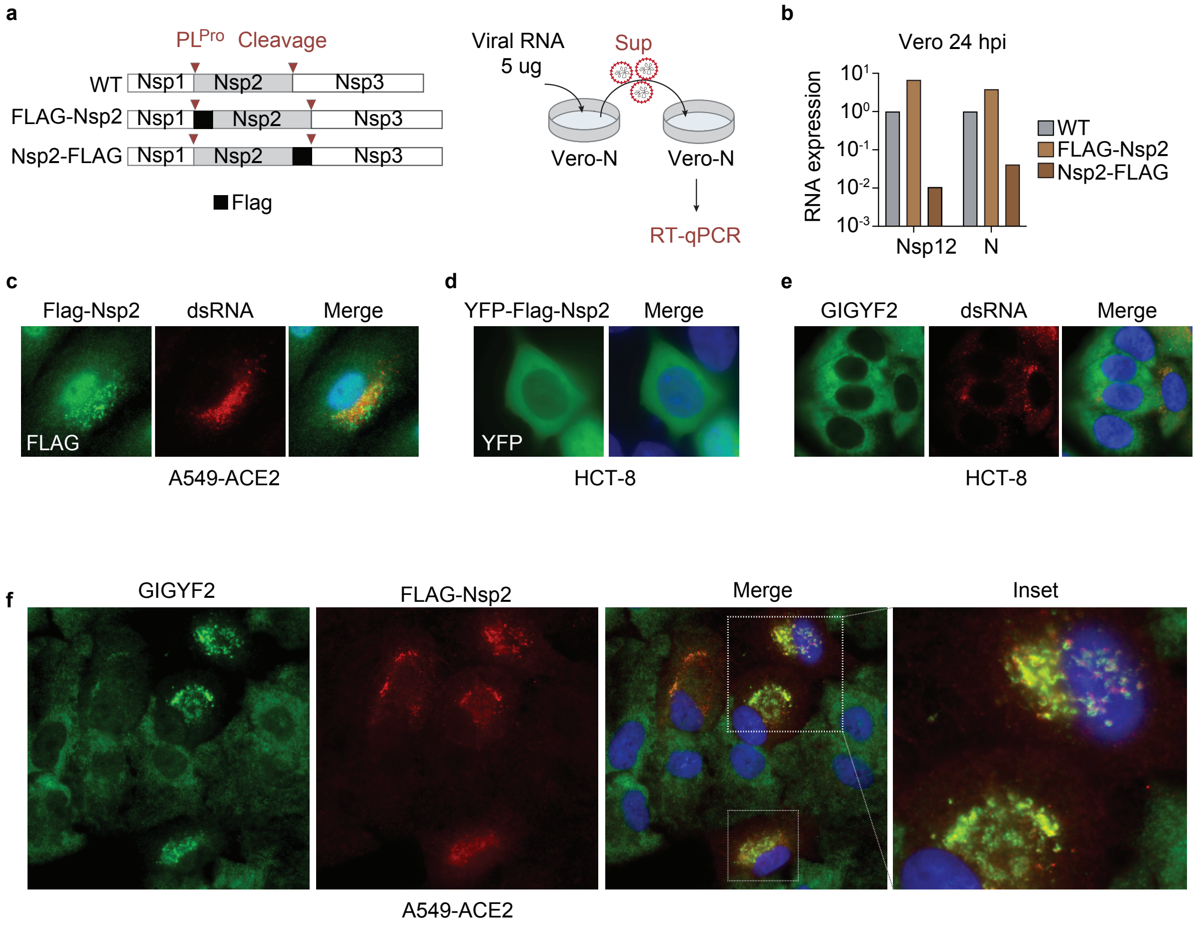

Supplementary Figure 3. Subcellular localization of GIGYF2 and Nsp2

(a) Schematic of FLAG-Nsp2 SARS-CoV-2 generation. Left: The N-terminal region of the Orf1 polyprotein of SARS-CoV-2 expressing WT, FLAG-Nsp2 (N-terminal FLAG) or Nsp2-FLAG (C-terminal FLAG). Proteolytic cleavage sites are indicated by arrowheads, with Nsp2 and FLAG colored gray and black, respectively. Right: Viral RNA (5 μg) was transfected into Vero-N cells. At 3 days post infection, the supernatant was used to infect fresh Vero-N cells. RNAs were extracted at 24 hpi and subjected to RT-qPCR.

(b) Relative RNA levels of Nsp12 and N following infection with SARS-CoV-2 expressing WT or FLAG-Nsp2 (C-terminal and N-terminal). RNAs were extracted from Vero-N cells at 24 hpi and subjected to RT-qPCR (n=1).

(c) Subcellular localization of Nsp2 after FLAG-Nsp2 SARS-CoV-2 infection. A549-ACE2 cells were infected with SARS-CoV-2 expressing FLAG-Nsp2 (MOI = 1). Immunofluorescence staining of FLAG (green) and dsRNA (red) was performed at 24 hpi along with DAPI staining.

(d) Subcellular localization of exogenously expressed SARs-CoV-2 YFP-FLAG-Nsp2 in HCT-8 cells. Images were obtained using the YFP signal, along with DAPI staining (blue).

(e) Subcellular localization of GIGYF2 upon HCoV-OC43 infection. HCT-8 cells were infected with HCoV-OC43 (MOI = 5). Immunofluorescence staining of GIGYF2 (green) and dsRNA (red) was performed at 24 hpi, along with DAPI staining (blue).

(f) Subcellular localization of GIGYF2 and Nsp2 following infection with SARS-CoV-2 expressing FLAG-Nsp2. A549-ACE2 cells were infected with SARS-CoV-2 expressing FLAG-Nsp2 (MOI = 10). Immunofluorescence staining of GIGYF2 (green) and FLAG (red) was performed at 6 hpi, along with DAPI staining (blue). The lower dashed frame area in the merged figure corresponds to the region shown in Figure 3b.

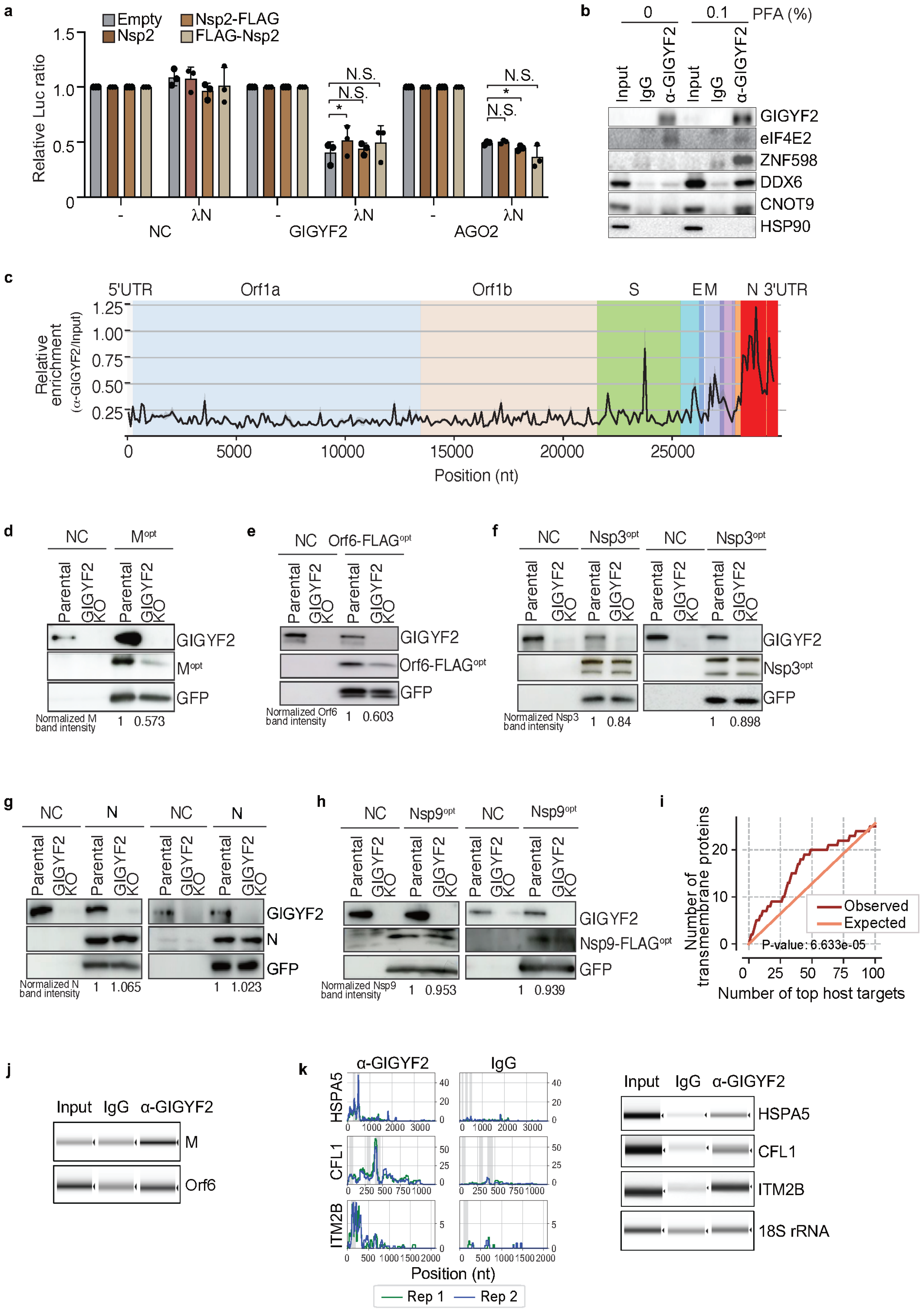
a
Empty
Nsp2
Nsp2-FLAG
FLAG-Nsp2
Relative Luc ratio
1.5
1.0
0.5
0
N.S.
N.S.
*
λN
NC
GIGYF2
AGO2
b
0
0.1
PFA (%)
Input
IgG
α-GIGYF2
GIGYF2
eIF4E2
ZNF598
DDX6
CNOT9
HSP90
c
5'UTR
Orf1a
Orf1b
S
E M
N 3'UTR
Relative enrichment (α-GIGYF2/Input)
1.25
1.00
0.75
0.50
0.25
0
5000
10000
15000
20000
25000
Position (nt)
d
NC
Mopt
Parental
GIGYF2 KO
GIGYF2
Mopt
GFP
Normalized M band intensity
1 0.573
e
NC
Orf6-FLAGopt
GIGYF2
Orf6-FLAGopt
GFP
Normalized Orf6 band intensity
1 0.603
f
NC
Nsp3opt
GIGYF2
Nsp3opt
GFP
Normalized Nsp3 band intensity
1 0.84
1 0.898
g
NC
N
GIGYF2
N
GFP
Normalized N band intensity
1 1.065
1 1.023
h
NC
Nsp9opt
GIGYF2
Nsp9-FLAGopt
GFP
Normalized Nsp9 band intensity
1 0.953
1 0.939
i
Number of transmembrane proteins
Number of top host targets
Observed
Expected
P-value: 6.633e-05
j
Input
IgG
α-GIGYF2
M
Orf6
k
α-GIGYF2
IgG
HSPA5
CFL1
ITM2B
Position (nt)
Rep 1
Rep 2
Input
IgG
α-GIGYF2
HSPA5
CFL1
ITM2B
18S rRNA

Supplementary Figure 4. Validation of GIGYF2 targets

(a) Tethering reporter assay assessing the function of GIGYF2. HEK293T cells were transfected with a combination of plasmids: 1) plasmids expressing GIGYF2 with or without λN fused to its C-terminus, 2) dual luciferase reporter plasmids expressing both firefly luciferase (Fluc) mRNAs with 9 repeats of Box B sites and control renilla luciferase (Rluc), and 3) empty (−) or SARS-CoV-2 Nsp2 plasmids with no tag, an N-terminal FLAG tag (FLAG-Nsp2), or a C-terminal FLAG tag (Nsp2-FLAG). Plasmids expressing only λN or AGO2 protein were used as negative (NC) and positive controls, respectively. Results are normalized to λN-deficient samples for each protein and shown as mean ± s.d. (n = 3). *P < 0.05, **P < 0.01, Two-sided Student's t-test.

(b) Co-immunoprecipitation of GIGYF2 and its known cofactors. HEK293T cells were crosslinked for 10 min with 0.1% of formaldehyde. Endogenous proteins were immunoprecipitated using either IgG or anti-GIGYF2 antibodies, followed by western blot analysis. 1% of input lysates was loaded along with the pulldown samples. Hsp90 was used as a negative control.

(c) Enrichment of GIGYF2 fCLIP-seq reads across the SARS-CoV-2 genome (bin size = 100 nt, shift = 100 nt). Different genomic regions are color-coded, with regions showing significant peak enrichment highlighted in red. The line and shadow indicate the mean enrichment (anti-GIGYF2/Size-matched input) and the standard deviation, respectively (n = 2).

(d-g) Expression changes of potential GIGYF2 targets upon GIGYF2 depletion. The expression levels of potential GIGYF2 targets (M (d), Orf6-FLAG (e), Nsp3 (f), N (g)) and GFP were detected by western blot analysis. The band intensities of the GIGYF2 targets were measured using ImageJ software and normalized to GFP. The superscript "Opt" denotes proteins with optimized codon sequences.

(h) Expression change of GIGYF2 non-target upon GIGYF2 depletion. The expression levels of a non-target protein (Nsp9-FLAG) and GFP were detected by western blot analysis. The band intensity of the non-target protein was measured using ImageJ software and normalized to GFP. The superscript "Opt" denotes proteins with optimized codon sequences.

(i) The enrichment of transmembrane protein-coding genes among the top-ranked GIGYF2-interacting host transcripts. One-sided KS test.

(j) Validation of GIGYF2 viral targets through RT-PCR following fCLIP without RNase treatment. The fCLIP RT-PCR products were analyzed using Tapestation.

(k) Validation of GIGYF2 host targets through RT-PCR following fCLIP without RNase treatment. Left: Gene-specific fCLIP-seq enrichment peaks of host targets. Green and blue lines indicate replicates, respectively. Right: The fCLIP RT-PCR products were analyzed using Tapestation. 18S rRNA serves as a loading control.
